# Behavioral and neurophysiological abnormalities during cued continuous performance tasks in patients with mild traumatic brain injury

**DOI:** 10.1002/brb3.966

**Published:** 2018-04-15

**Authors:** Weixiang Zhao, Ruhong Wu, Suhong Wang, Haihui Qi, Yitao Qian, Suinuan Wang

**Affiliations:** ^1^ Department of Neurosurgery The Third Affiliated Hospital of Soochow University Changzhou Jiangsu China; ^2^ Department of Neuroscience The Third Affiliated Hospital of Soochow University Changzhou Jiangsu China

**Keywords:** conflict monitoring, mild traumatic brain injury, response inhibition, sustained attention

## Abstract

**Objective:**

This study's aim was to investigate the features and neural mechanisms of sustained attention in patients with mild traumatic brain injury (mTBI) by comparing and analyzing neuropsychological, behavioral, event‐related potentials, and event‐related desynchronization and synchronization between mTBI patients and healthy controls.

**Methods:**

Twenty mTBI patients with mTBI and 20 healthy controls underwent the Mini‐Mental State Examination (MMSE) and a cued continuous performance task (AX‐CPT). Neuropsychological, behavioral, and electroencephalogram (EEG) data were collected and analyzed.

**Results:**

There were significant differences between the mTBI group and the control group in their MMSE total scores, attention, and calculation, but there were no significant differences in orientation, memory, recall, and verbal scores. There were significant differences between the mTBI group and the control group in hitting the number, reaction time, and the number of errors of omission, but there were no significant differences in the number of false errors. The amplitude of Go‐N2 and Nogo‐N2 was significantly smaller for the mTBI group than that for the control group. The amplitude of Go‐P3 was significantly smaller for the mTBI group than that for the control group, but not for the amplitude of Nogo‐P3. The Go‐αERS were significantly less for the mTBI group than for the control group during the 0–200 ms after the stimulus onset. The Go‐αERD and Nogo‐αERD were significantly less for the mTBI group than for the control group during the 600–1,000 ms after the stimulus onset. The Go‐βERS were significantly less for the mTBI group than for the control group during the 200–400 ms after the stimulus onset. There were no significant differences in the Nogo‐αERS and Nogo‐βERD/ERS between the mTBI group and the control group.

**Conclusion:**

Patients with mTBI exhibited impairments in sustained attention and conflict monitoring, while response inhibition may have been spared.

## 1 INTRODUCTION

Traumatic brain injury (TBI) is the leading cause of morbidity and mortality in adults in highly developed countries, with the highest incidence in men 15–24 years of age (Shumskaya, Andriessen, Norris, & Vos, 2012). The vast majority (75%–90%) of TBIs are considered to be mild in nature (Zhou et al., 2012). In China, common causes of mild TBI (mTBI) include traffic accidents and falls. Many mTBI patients exhibit varying degrees of physical, cognitive, and psychological dysfunction. Specifically, research investigating cognitive function after mTBI has shown that almost every patient exhibits varying features of cognitive disorder, such as deficits in attention, memory, and reasoning, and a decline in mental acuity and information processing speed (Dikmen, McLean, Temkin, & Wyler, 1986; Rabinowitz & Levin, 2014). Consequently, these cognitive disorders have a profound effect on patients and their families. Cognitive impairments caused by TBI affect work, relationships, and the activities of daily life, which are difficult to quantify in terms of personal and economic losses.

Mild traumatic brain injury (mTBI) frequently causes attention impairment, which can significantly affect recovery and rehabilitation from injury (Schmitter‐Edgecombe & Robertson, 2015). The most prevalent impairments to attention after TBI include processing speed, attentional capacity, sustained and selective attention, and supervisory attentional control (Dymowski, Ponsford, & Willmott, 2016; Mathias & Wheaton, 2007). Studies have shown that attentional function can be a prognostic indicator in TBI patients.

At the same time, understanding the features and neural mechanisms of attention is also important to recovery and rehabilitation after suffering from TBI. Conflict monitoring and response inhibition are the two main areas of attention, especially in states of sustained attention. Nonetheless, research investigating conflict monitoring and response inhibition in mTBI patients has been scarce, primarily because the basic clinical neuroimaging findings in the majority of patients with mTBI are normal (Jacobs et al., 2010; Smits et al., 2007). However, neuropsychological assessment scales and temporally sensitive electroencephalography (EEG)‐based methods can reveal subtle cognitive disorders. Accordingly, in this study, we used the Mini‐Mental State Examination (MMSE) and the cued continuous performance task (AX‐CPT) to explore the features and neural mechanisms of impaired sustained attention in patients with mTBI. Neuropsychological, behavioral, and EEG data were collected and analyzed.

The MMSE has been widely used in the screening of cognitive impairment in patients with TBI. Its reliability and validity have been tested in previous studies (Wen, Zhang, Niu, & Li, 2008), and it can be completed relatively quickly. In this study, we applied the Chinese version of the MMSE questionnaire to measure cognitive function in patients with mTBI while collecting EEG data. The CPT task was first developed by Beck and Rosvold in 1956 and is used to test response inhibition in patients with TBI. The CPT task can test abilities of inhibition, execution, alertness, and short‐term memory and has become an experimental paradigm commonly used in research investigating attention and working memory. Halperin (Halperin, McKay, & Newcorn, 2002) used the AX‐CPT to detect inhibition of sustained attention and impulsivity inhibition.

This study used two quantitative EEG methods to explore the neural mechanisms of attention and executive function disorders in patients with mTBI—event‐related potentials (ERPs) and event‐related desynchronization and synchronization (ERD/ERS).

Event‐related potential is closely related to the neurological activity of the cerebral cortex. When an event takes place, time‐locked changes can be induced in the activity of the neuron group, and these changes are measured as ERPs.

Event‐related potentials can reveal changes in associated nerve activity when individuals exhibit a behavior, such as selective attention or inhibition control (Johnstone, Barry, & Clarke, 2013). Recent ERP studies reported that the amplitude and latency of some of the ERP components of TBI patients were abnormal compared with healthy controls (Larson, Kaufman, Schmalfuss, & Perlstein, 2007; Segalowitz, Dywan, & Unsal, 1997). Research investigating visual and auditory attention function in TBI patients found that in visual attention tests, TBI patients showed decreased N2b amplitude, while in hearing tests they showed decreased N2b amplitude, extended N2b, and P3 latency (Duncan, Summers, Perla, Coburn, & Mirsky, 2011; Duncan et al., 2009). We focused specifically on the N2 and P3 of the ERP components and examined differences in the N2 and P3 of the ERP components between patients with mTBI and healthy controls.

Event‐related desynchronization and ERS indicate decreases or increases in power within a specific frequency band when an event takes a place. Specifically, ERD indicates that nervous activity in a specific frequency band is desynchronized with others, resulting in reduced rhythmic activity and increased cortical activity. On the other hand, ERS represents the opposite activity. The nervous activity in a specific frequency band is synchronized with others, resulting in increased rhythmic activity and reduced cortical activity (Lee, Lindquist, & Nam, 2017; Nam, Jeon, Kim, Lee, & Park, 2011). These processes are relevant to the neural mechanisms of attention and executive function, and we predict that ERD/ERS will be different between patients with mTBI and healthy controls.

The ERD/ERS calculation method is based on the time–frequency energy distribution, calculating the time–frequency power distribution matrix of the signal in the resting state and the induced state after using the Morlet wavelet to transform of the same length of the resting state and the induced signal. The advantage of this calculation method is that it is very intuitive, showing different time points and different frequencies of ERD/ERS value and thus revealing the law of power changes over time and frequency (Zygierewicz, Durka, Klekowicz, Franaszczuk, & Crone, 2005).

Both the ERD and the ERS are typically measured in five major frequency bands, the delta (0–4 Hz), theta (4–8 Hz), alpha (8–13 Hz), beta (13–30 Hz), and gamma bands (30–200 Hz; Uhlhaas, Haenschel, Nikolic, & Singer, 2008). We focused specifically on the ERD/ERS of the alpha‐ and beta‐band power and examined differences in the ERD/ERS of the alpha‐ and beta‐band power between patients with mTBI and healthy controls.

## 2 MATERIALS AND METHODS

### 2.1 Participants

Twenty patients with mTBI were recruited from the Department of Neurosurgery of The Third Affiliated Hospital of Soochow University between January 2014 and February 2016. Inclusion criteria were as follows: (i) meet the diagnostic criteria established by the American Association of Rehabilitation Medicine of mTBI (Kay et al., 1993), that is, a hospital admission Glasgow Coma Scale (GCS) score of 13–15, with or without loss of consciousness (LOC) for 30 min and with or without posttraumatic amnesia (PTA) for 24 hr; (ii) between 2 and 28 days postinjury, CT imaging was almost normal; (iii) age 20 years to 55 years; (iv) education of more than 6 years; (v) all participants are right‐handed; (vi) all patients’ hearing and vision (or corrected vision) are normal, and there is no history of mental illness, neurological diseases, or history of alcohol or drug abuse. Patients with mTBI were age‐, gender‐, and education‐matched to 20 healthy control subjects (Table 1). All participants were asked to participate in the MMSE. The study was approved by the Ethics Committee of the Third Affiliated Hospital of Soochow University, and all participants gave written informed consent.

**Table 1:** Demographics and injury characteristics of patients with mTBI and control subjects

	mTBI	Control	*p*
Demographics			
Age (years)	41.9 ± 9.2	41.2 ± 8.9	.8
Male/female	15:5	15:5	
Education (years)	10.1 ± 3.7	10.4 ± 3.8	.8
Time to test from injury (days)	15.8 ± 6.2		
Injury characteristics			
GCS score, *n* (%)			
15	16 (80)		
14	3 (15)		
13	1 (5)		
LOC, *n* (%)	9 (45)		
PTA, *n* (%)			
No	13 (65)		
1–30 min	5 (25)		
>30 min	2 (10)		

GCS, Glasgow Coma Scale; LOC, loss of consciousness; mTBI, mild traumatic brain injury; PTA, posttraumatic amnesia.

### 2.2 Neuropsychological assessment

The MMSE was initially developed to evaluate patients with mild cognitive impairment (Lee, Koh, Moon, Park, & Song, 2015), and it was one of the most influential cognitive disorders screening tool ever developed. In this study, all participants used the Chinese version of the MMSE first. The MMSE has 30 items. We divided it into five aspects to analyze it easily, including orientation, memory, attention and calculation, recall, and verbal. Decision criteria: The highest score is 30. The cognitive is normal when the score is between 27 and 30, while a disorder exists when the score is <27.

### 2.3 Experimental paradigm

**Figure 1:**
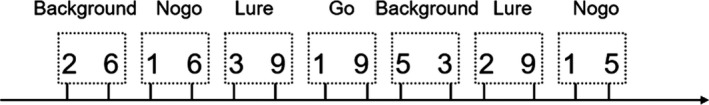
Participants were instructed to press the key using their right index finger only when the numeral 1 was directly followed by the numeral 9. All other conditions, including the three conditions of Nogo, Lure, and Background, were to be ignored

The AX‐CPT included Go, Nogo, Lure, and Background four conditions embedded in a vigilance task with a pseudorandom sequence of 700 white Arabic numeral symbols (1, 2, 3, 4, 5, 6, 7, 8, and 9) presented in the center of a black screen (Figure 1). Every numeral was presented for 200 ms, separated by a 1,200‐ms blank screen. The numeral 1 served as a cue, initiating a Go‐Nogo task and inducing response preparation. Participants were instructed to press a button with the index finger of their right hand as fast as possible when the numeral 1 was followed directly by the numeral 9 (Go condition, 20% probability), but they had to withhold response to the numeral 1 when it was not followed by 9 (Nogo condition, also 20% probability). Moreover, the single 9 preceded by a number other than 1 (Lure condition, 20% probability) also required no response. A total of 140 numeric sequences involving neither the numeral 1 nor the numeral 9 (Background condition, 40% probability) were presented. Participants were instructed to press the button as quickly and accurately as possible. Before formally starting the experiment, participants were allowed to practice in the laboratory, until they could control the experimental task completely. To avoid experimental fatigue impact for the participants, the experiment was divided into two sessions, and there was a 3‐min break between each session.

### 2.4 Data acquisition and signal processing

The experiment was programmed and executed with E‐Prime 2.0 (http://scicrunch.org/resolver/SCR_009567) software (Psychology Software Tools Inc., Pittsburgh, PA, USA). EEG was recorded with a 128‐channel EGI Geodesic Sensor Net connected to a DAC‐coupled high input impedance amplifier. Initial offline processing of the data was performed using Net Station software (version 4.3.1; EGI). The sampling rate was 500 Hz, with 0.3–30 Hz filters. Individual sensors were adjusted until impedance was less than 50 KΩ for all sensors. CZ was reference electrode. The participant was made comfortable in a chair with a 128‐channel electrode cap in an acoustically shielded and dimly lit room. Stimuli were displayed on a monitor at 80 cm distance from the participant's eyes, with 0.7° of visual angle horizontally and 1.4 vertically. The right index finger was placed on a prefixed button.

Electroencephalogram data were preprocessed using the EEGLAB toolkit (http://scicrunch.org/resolver/SCR_007292) based on the MATLAB (http://sccn.ucsd.edu/eeglab) (Delorme et al., 2011). After data acquisition was completed, epochs were constructed from 200 to 1,000 ms relative to stimulus onset (where stimulus onset time was 0 ms), epochs were classified according to Go, Nogo, Lure, and Background four conditions, and the data channels were located on EEGLAB. Data were refiltered of 0.3–30 Hz. Epochs were then baseline‐corrected relative to −100 to 0 ms. Trials containing eyeblink, eye movements, or muscle movements were removed using ADJUST (an automatic EEG artifact detector) combined with artificial screening method (Mognon, Jovicich, Bruzzone, & Buiatti, 2011).

### 2.5 Data analysis

Data were analyzed using Brainstorm 3.2 (http://scicrunch.org/resolver/SCR_001761), a documented program that is available for free download online under the GNU general public license (http://neuroimage.usc.edu/brainstorm). ERPs were computed for superimposed average and total average. Here, we choose to compare the two group ERPs in the Go and Nogo conditions and to measure the latency and amplitude of the N2 in FZ and the P3 in PZ. The measuring time window of the N2 and P3: N2, 150–250 ms; P3, 400 ms–600 ms.

We made a copy of the ICBM152 anatomy and set it as the default for the study (Fonov et al., 2011). This means that we will be able to use this template brain as a substitute for the subjects without an individual MRI or as the common brain for group analysis. The Mindboggle (http://mindboggle.info) atlas of FreeSurfer was adopted to apply anatomical labels (Klein & Tourville, 2012).

The length of epoch was 1,200 ms (from −200 to 1,000 ms). The second 100‐ms interval was the reference, while the next 1,000 ms was the interval of interest. The power of 1,000 ms was averaged for five time bins with a length of 200 ms each. The study compute time–frequency decomposition is based on the convolution of the signal with a series of complex Morlet wavelets. ERD and ERS were described by time–frequency maps divided by a spectrum of the baseline state (Pfurtscheller & Lopes da Silva, 1999), which was selected in time intervals of 100 ms before stimuli.

ERD, ERS = (*A*−*R*)**/** *R* × 100%, *R* is the average power during the reference period (i.e., from −100 to 0 ms), and *A* is the average power in the interval of interest (i.e., 0–1,000 ms).

### 2.6 Statistical analysis

Single factor ANOVA was used to analyze the neuropsychological and behavioral data. The repeated measures ANOVA and independent samples *t* test were used to analyze the EEG data, with condition (Go and Nogo) as a within‐subjects factor and group (mTBI and normal control) as a between‐subjects factor. The statistical results were expressed as $\overset{¯}{x}$ ± *s*. Differences were considered significant if the *p*‐value was <.05. Based on the statistics of ERD/ERS, we examined the correlation between the ERD/ERS of some regions and the behavioral results.

## 3 RESULTS

### 3.1 Neuropsychological results

There were significant differences between the mTBI group and the control group in total score and attention and calculation (*p *<* *.05), but there were no significant differences in orientation, memory, recall, or verbal scores (*p *>* *.05; Table 2).

**Table 2:** Neuropsychological results

Group	Total score	Orientation	Memory	Attention and calculation	Recall	Verbal
mTBI	28.00 ± 1.18	9.90 ± 0.30	2.95 ± 0.22	3.95 ± 0.59	2.75 ± 0.43	8.45 ± 0.59
Control	29.20 ± 1.03	10.00 ± 0.01	3.00 ± 0.01	4.65 ± 0.48	2.80 ± 0.40	8.75 ± 0.43
*t*	−3.16	−1.37	−0.94	−3.79	−0.35	−2.21
*p*	.003**	.18	.36	.001**	.73	.03*

mTBI, mild traumatic brain injury.

Data presented as mean ± *SD* unless otherwise indicated.

*,**Statistically significant.

### 3.2 Behavioral results

There were significant differences between the mTBI group and the control group in mean hitting number, the number of errors of missing, and reaction time (*p *<* *.05). There was no significant difference in the number of false errors between the two groups (*p *>* *.05; Table 3).

**Table 3:** Behavioral results

	Hitting number	Errors of missing	Reaction time, ms	False errors
mTBI	66.76 ± 3.26	3.23 ± 3.27	532.57 ± 129.53	0.36 ± 1.01
Control	69.11 ± 1.41	0.89 ± 1.42	412.61 ± 116.264	0.52 ± 0.88
*t*	−2.710	2.756	2.842	−0.492
*p*	.011*	.010*	.008**	.626

mTBI, mild traumatic brain injury.

Data presented as mean ± *SD* unless otherwise indicated.

*,**Statistically significant.

### 3.3 Event‐related potential results

**Figure 2:**
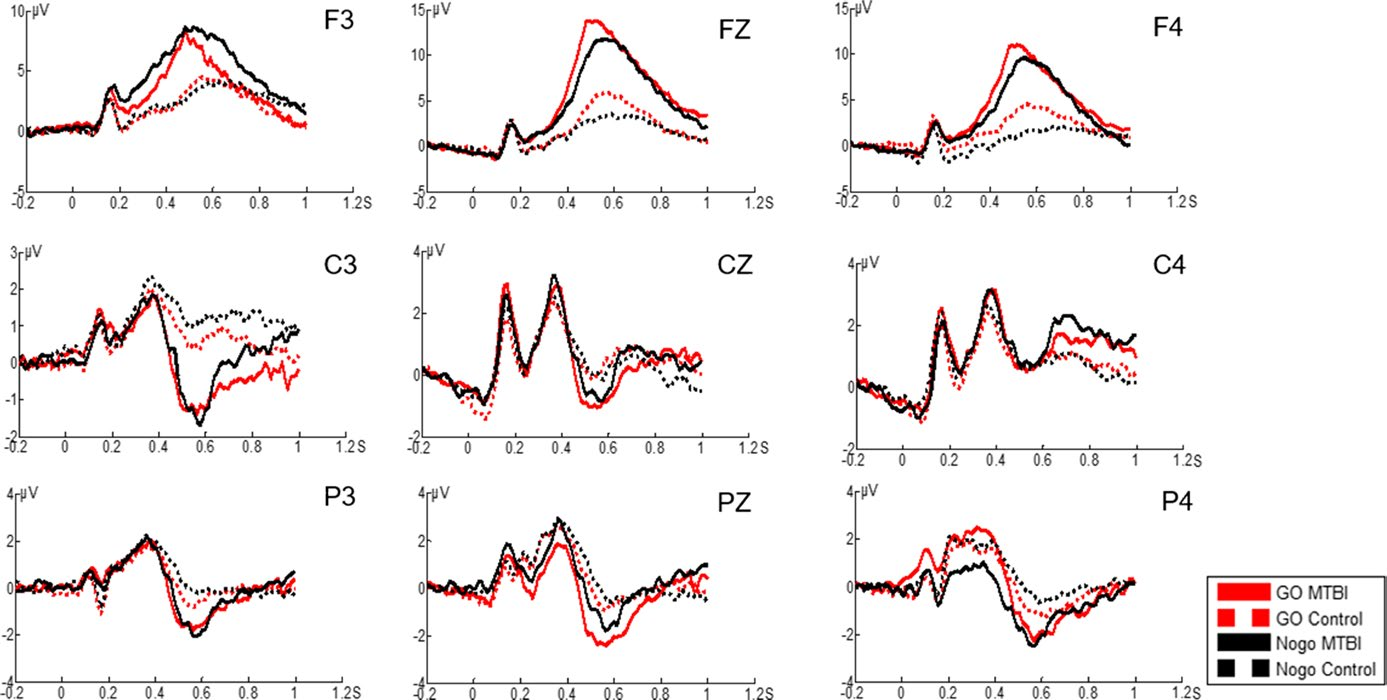
The figure shows the grand average event‐related potentials of the mild traumatic brain injury (mTBI) group and the control group under the conditions of “Go” and “Nogo”

Nine channels were selected to observe ERPs (Figure 2). FZ and PZ were chosen to compare the differences between the mTBI group and the control group.

### 3.4 ANOVA for N2, P3 amplitude, and latency

The main effect of group was significant for N2 amplitude (*p *<* *.05), but the main effect of condition and the interaction effect were not significant (*p *>* *.05). Group and condition had no significant main effect or interaction effect on the latency of N2 (*p *>* *.05). Although the main effect of group and condition were significant for P3 amplitude (*p *<* *.05), the interaction effect was not significant (*p *>* *.05). Group and condition had no significant main effect and interaction effect on the amplitude of P3 (*p *>* *.05).

### 3.5 Comparison of the N2 and P3 amplitude between the mTBI and control groups

The amplitude of Go‐N2 and Nogo‐N2 was significantly smaller for the mTBI group than that for the control group (*p *<* *.05). The amplitude of Go‐P3 was significantly smaller for the mTBI group than that for the control group (*p *<* *.05), while not significantly smaller for Nogo‐P3 (*p *>* *.05; Table 4).

**Table 4:** Comparison of N2 and P3 amplitude between the mTBI group and the control group

Component	Condition	mTBI group	Control group	*T*	*p*
N2	Go	0.69 ± 0.65	1.28 ± 0.91	−2.175	.037*
	Nogo	0.5 5 ± 0.74	1.25 ± 0.98	−2.350	.025*
P3	Go	1.51 ± 0.88	2.33 ± 1.07	−2.440	.020*
	Nogo	2.24 ± 0.59	2.53 ± 0.60	−1.421	.165

mTBI, mild traumatic brain injury.

*,**Statistically significant.

### 3.6 ERD/ERS results

**Figure 3:**
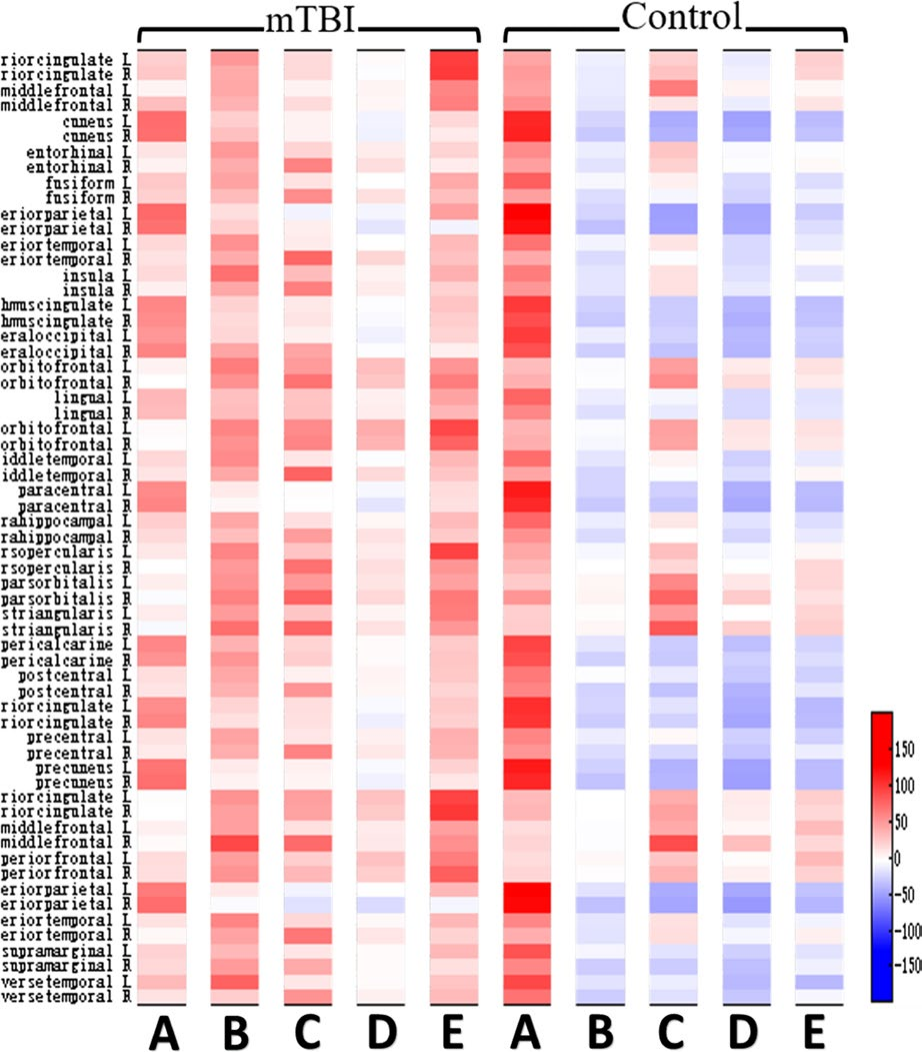
Brainarea power maps of ERS/ERD in the alpha band (8–13 Hz) during the Go‐Nogo task show statistical differences between mild traumatic brain injury patients and healthy controls during five time periods: (a: 0–200 ms, Go task); (b: 800–1,000 ms, Go task); (c: 400–600 ms, Nogo task); (d: 600–800 ms, Nogo task); and (e: 800–1,000 ms, Nogo task)

**Figure 4:**
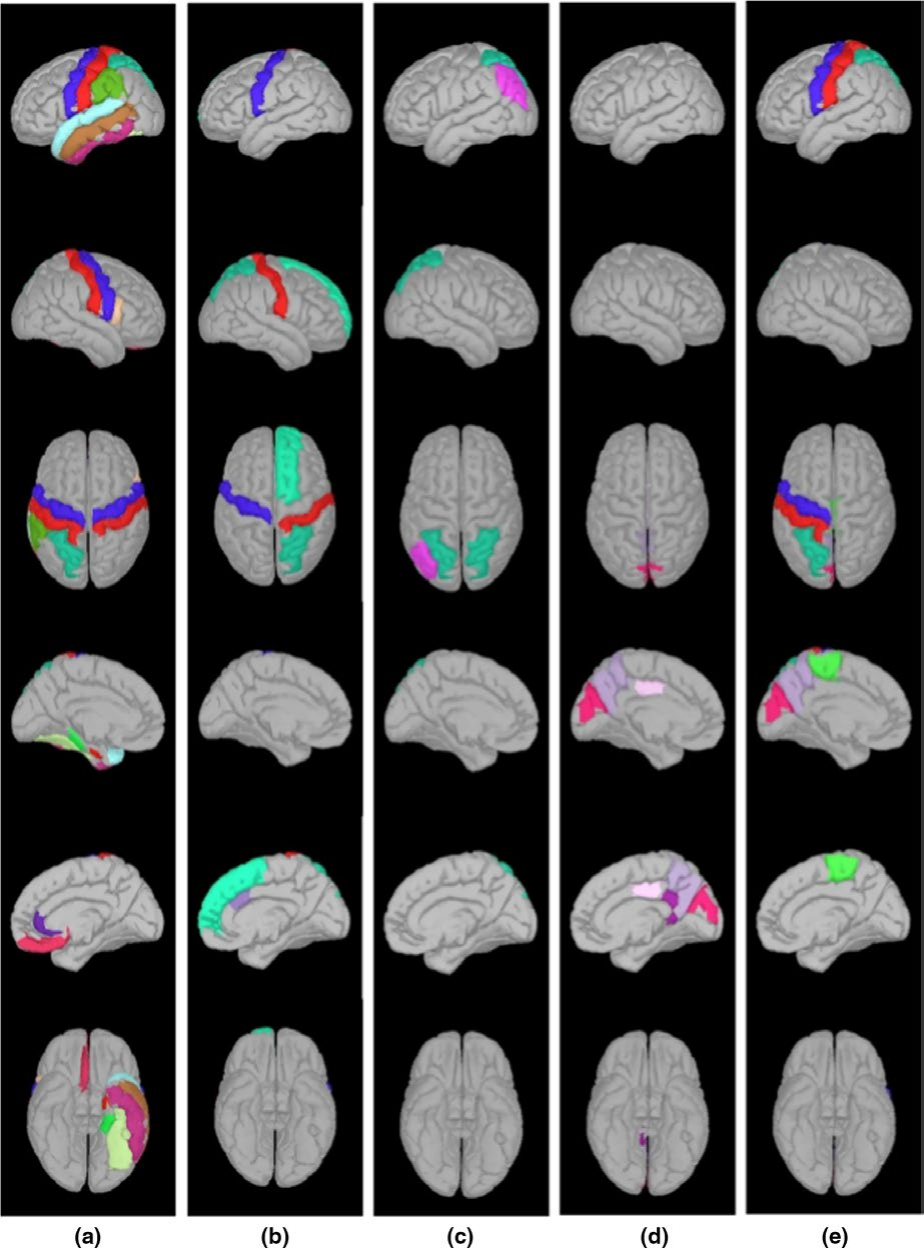
The source maps show statistically significant brain regions during five time periods marked with different colors: (a: 0–200 ms, Go task); (b: 800–1,000 ms, Go task); (c: 400–600 ms, Nogo task); (d: 600–800 ms, Nogo task); and (e: 800–1,000 ms, Nogo task)

**Figure 5:**
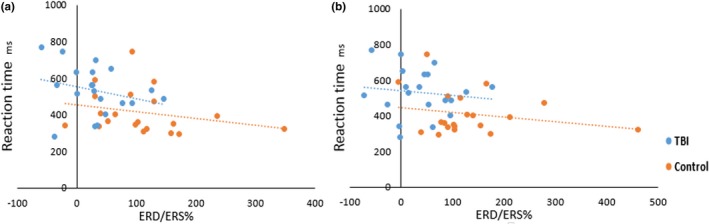
The linear trend figures show correlation between reaction time and alpha‐band power of Go during the 0–200 ms after the stimulus onset. (a: the left inferior temporal); (b: the left supramarginal)

We first analyzed the ERD/ERS of the alpha‐ and beta‐band power of Go event. As shown in Figure 3, Brainarea power maps of ERS/ERD in the alpha band (8–13 Hz) during the Go‐Nogo task show statistical differences between mTBI patients and healthy controls during five time periods. The ERS in the mTBI group was decreased in the regions as described in Figure 4a (*p *<* *.05), as compared to the control group during the 0–200 ms after the stimulus onset. Compared to the control group during the 600–800 ms after the stimulus onset, we found that the mTBI group showed less αERD only in the right inferior temporal regions (*t* = 2.431, *p *<* *.05). αERD in the mTBI group was decreased in the regions as described in Figure 4b (*p *<* *.05), as compared to the control group during the 800–1,000 ms after the stimulus onset. At beta band (13–30 Hz), ERS in the mTBI group was decreased in both the right lingual and the right pericalcarine regions (*p *<* *.05), as compared to the control group during the 200–400 ms after the stimulus onset. However, βERS in the mTBI group was increased in the superior frontal region (*p *<* *.05), as compared to the control group during the 800–1,000 ms after the stimulus onset. Then, we analyzed the ERD/ERS of the alpha‐ and beta‐band power of Nogo. At alpha band (8–13 Hz), as compared to the control group, ERD in the mTBI group was decreased in the regions as described in Figure 4c (*p *<* *.05) during the 400–600 ms after the stimulus onset and Figure 4d (*p *<* *.05) during the 600–800 ms after the stimulus onset. During the 800–1,000 ms after the stimulus onset, the mTBI group showed more ERS, and the control group showed more ERD in the regions as described in Figure 4e. At beta band (13–30 Hz), there were no significant differences in ERD and ERS between the mTBI group and the control group. In Figure 5, the left inferior temporal (*p *<* *.01) and the left supramarginal (*p *<* *.01) during the 0–200 ms after the stimulus onset were selected to observe the correlation between the alpha‐band power of Go with reaction time, and there is an inverse trend between them.

## 4 DISCUSSION

The present study was the first to explore the neural mechanisms of sustained attention and executive function disorders in TBI patients using 128‐channel high‐density EEG technology while participants completed the AX‐CPT. For each of the neuropsychological, behavioral, and EEG findings, we discuss whether findings were consistent with our a priori hypotheses and offer preliminary interpretations of our findings.

We first applied the MMSE to measure cognitive function in mTBI patients and then explored the characteristics of sustained attention. In the MMSE questionnaire, individuals in the mTBI group scored significantly lower than the control group in total score, attention, and calculation. No significant differences, however, were evident in orientation, memory, recall, or verbal scores. The neuropsychological results of our study suggest that the mTBI group experienced mild cognitive disorders, especially in attention and calculation.

The AX‐CPT has a high sensitivity for the detection of sustained attention deficit after TBI. In this study, the probability in the target tasks was lower than background; the repetition stimulus was less frequent and was rapid. Subjects were required to observe a number and then to respond quickly to low‐probability events. Subjects were required to maintain an alert state, while simultaneously needing high‐response efficiency to complete the continuous task effectively. Studies have suggested that continuous changes in stimulus and the cumulative effect of continuous persistence can adjust the repetitive inhibition effect (Olofsson & Polich, 2007; Ranganath & Rainer, 2003).

The hitting number is associated with sustained attention, the number of errors of omission can indicate the degree of attention deficit, the number of false errors reflects executive control and impulse inhibition, and reaction time is related to the reaction rate (Erdodi, Roth, Kirsch, Lajiness‐O'neill, & Medoff, 2014). In this study, there were significant differences between the mTBI group and the control group in mean hitting number, the number of errors of missing, and reaction time (*p *<* *.05), and there was no significant difference in the number of false errors between the two groups (*p *>* *.05). Thus, the behavioral results of our study suggest that the mTBI group had significant impairments in sustained attention and reaction speed, while response inhibition was spared, which is consistent with the neuropsychological results. In previous studies that have used the reaction time task to explore mechanisms of sustained attention deficit, TBI patients performed significantly poorer than healthy controls in accurately completing tasks (Bonnelle et al., 2011). Results of the current study further suggest that patients with mTBI experience impairments in sustained attention and reaction speed.

The ERP results demonstrated significantly smaller amplitudes for Go‐N2, Go‐P3, and Nogo‐N2 in the mTBI group than the control group, but not in the amplitude for Nogo‐P3. Recent studies suggest that patients with severe TBI exhibit lower amplitude and longer latency (Larson, Clayson, & Farrer, 2012). Studies investigating the rehabilitation of TBI patients found that, with improved cognitive function, TBI patients exhibited significantly increased ERP amplitudes with shorter latencies (Iwanaga, Kato, Okazaki, & Hachisuka, 2015). In the current study, we demonstrated that the ERPs of mTBI patients were abnormal. The distribution of attention resources, the process of conflict monitoring, and the response inhibition are the important parts of attention, especially to sustained attention. Previous research using the AX‐CPT combined with ERPs suggested that Go‐N2 and Go‐P3 were related to the distribution of attention resources, Nogo‐N2 was related to the process of conflict monitoring, and Nogo‐P3 primarily reflected the response inhibition function (Guan et al., 2015; Nicholls, Bruno, & Matthews, 2015). Our study showed that patients with mTBI exhibited impairments in sustained attention, maybe due to the impairments of the distribution of attention resources and the process of conflict monitoring, sustained attention, and conflict monitoring, while response inhibition may have been spared. Furthermore, because the Nogo response requires reaction inhibition, and the N2 components of the Go and Nogo conditions are different, only N2 can be elicited (Duncan et al., 2009). However, other studies have suggested that Nogo‐P3 is not closely related to response inhibition, while P3 was only related to the process of inhibition (Wu et al., 2015).

The Go‐αERS results demonstrated significantly less αERS in the mTBI group than the control group during the 0–200 ms after the stimulus onset. Previous research suggested that alpha synchronization reflects top‐down inhibitory control (Klimesch, Sauseng, & Hanslmayr, 2007), and we posit that this inhibition serves to reduce potential interference and allocates processing resources to the brain areas responsible for Go encoding (Palva & Palva, 2007). Alpha activity has been shown to reflect the disengagement of task‐irrelevant regions in attention tasks (Huang et al., 2013; Meeuwissen, Takashima, Fernandez, & Jensen, 2011; Poch, Campo, & Barnes, 2014). Our study showed that patients with mTBI exhibited impairments in sustained attention, maybe due to the impairments of the distribution of attention resources. The Go‐αERD results demonstrated significantly less αERD in the mTBI group than the control group during the 600–1,000 ms after the stimulus onset. The anticipatory ERD might reflect the preactivation of neural networks (Klimesch et al., 2007). Further explanation is the preactivation of neural networks reduced in the mTBI group. The Go‐βERS results demonstrated significantly less βERS in the mTBI group than the control group during the 200–400 ms after the stimulus onset. There is convincing evidence that the beta frequency range has been linked to cognitive processes and visual attention (Gross et al., 2004). The βERS between extrastriate areas was observed in intracranial recordings during maintenance of objects in short‐term memory (Tallon‐Baudry, Bertrand, & Fischer, 2001). In addition, βERS between temporal and parietal areas was evident in EEG recordings during object processing (von Stein, Rappelsberger, Sarnthein, & Petsche, 1999). If so, we can consider that patients with mTBI exhibited impairments in sustained attention, maybe due to the impairments of visual attention and short‐term memory. As for the differences in Nogo‐αERD between the mTBI group and the control group, the explanation is the same as for Go‐αERD that preactivation of neural networks reduced in the mTBI group. There were no significant differences in Nogo‐αERS and Nogo‐βERD/ERS between the mTBI group and the control group.

Research investigating sustained attention and response inhibition in TBI patients has been inconsistent, possibly because the response inhibition function is not a single structure but the outcome of multiple neural mechanisms. Therefore, disparities in experimental protocols, different severities of brain injury, and other factors will contribute to inconsistent results among studies.

In previous studies, the anterior cingulate cortex (ACC) and the prefrontal cortex (PFC) have been reported to play important roles in conflict monitoring and the control of executive function. The ACC can monitor ongoing tasks and, in the event of a conflict, provide the signal to configure resources to strengthen attention when the conflict load is increased. The PFC can also adjust attention by allocating resources effectively; therefore, the PFC may be a high‐level regulatory structure of attention networks (Bryden et al., 2016; Padrao, Rodriguez‐Herreros, Perez Zapata, & Rodriguez‐Fornells, 2015). Recent research suggests that the dorsolateral PFC (DLPFC), the ventrolateral PFC (VLPFC), and the presupplementary motor areas are particularly important to response inhibition in the Nogo condition. The main function of the ACC is to inhibit the conflict, while the DLPFC and the VLPFC may be the specialized response inhibition centers in the brain. However, when the difficulty of experimental paradigms is increased, the ACC appears to play a major role in response inhibition (Chikazoe, 2010). Studies involving functional magnetic resonance imaging suggest that the activation of related brain regions in the ACC and PFC in TBI patients with sustained attention disorders is abnormal (Ham et al., 2014; Mannarelli et al., 2015), which suggests an important association between the activation of the ACC and PFC. In the current study, the conflict monitoring disorder in mTBI patients may have been accompanied by abnormal activation of the ACC, while the spared response inhibition may be associated with related networks in the PFC with no obvious impairments. However, when the severity of brain injury or the difficulty of the tasks increase, the ACC may also play an important role and influence response inhibition.

In conclusion, the current study was the first to investigate the features and neural mechanisms of sustained attention in patients with mTBI with 128‐channel high‐density EEG technology. We analyzed and compared neuropsychological, behavioral, ERPs, and ERD/ERS of patients and healthy controls and demonstrated that patients with mTBIs experience impairments in sustained attention due to the impairments of the distribution of attention resources and conflict monitoring, with a possible sparing of response inhibition. This study provided some reference values for the evaluation of sustained attention disorder after TBI and helped us explore the pathogenesis of attention disorders following TBI. In future studies, a larger number of subjects with injuries to different parts of the brain with varying injury severity will be required to more extensively explore the neurological mechanisms of sustained attention disorder after TBI. This will provide more reference values for the diagnosis and rehabilitation of attention disorder in patients with TBI.

## CONFLICT OF INTERESTS

None declared.

## References

[brb3966-bib-0001] Bonnelle, V. , Leech, R. , Kinnunen, K. M. , Ham, T. E. , Beckmann, C. F. , De Boissezon, X. , … Sharp, D. J. (2011). Default mode network connectivity predicts sustained attention deficits after traumatic brain injury. The Journal of Neuroscience, 31(38), 13442–13451. https://doi.org/10.1523/JNEUROSCI.1163-11.2011 21940437 10.1523/JNEUROSCI.1163-11.2011 PMC6623308

[brb3966-bib-0002] Bryden, D. W. , Burton, A. C. , Barnett, B. R. , Cohen, V. J. , Hearn, T. N. , Jones, E. A. , … Roesch, M. R. (2016). Prenatal nicotine exposure impairs executive control signals in medial prefrontal cortex. Neuropsychopharmacology, 41(3), 716–725. https://doi.org/10.1038/npp.2015.197 26189451 10.1038/npp.2015.197 PMC4707818

[brb3966-bib-0003] Chikazoe, J. (2010). Localizing performance of go/no‐go tasks to prefrontal cortical subregions. Current Opinion in Psychiatry, 23(3), 267–272. https://doi.org/10.1097/YCO.0b013e3283387a9f 20308899 10.1097/YCO.0b013e3283387a9f

[brb3966-bib-0004] Delorme, A. , Mullen, T. , Kothe, C. , Akalin Acar, Z. , Bigdely‐Shamlo, N. , Vankov, A. , & Makeig, S. (2011). EEGLAB, SIFT, NFT, BCILAB, and ERICA: New tools for advanced EEG processing. Computational Intelligence and Neuroscience, 2011, 130714 https://doi.org/10.1093/arclin/acu026 21687590 10.1155/2011/130714 PMC3114412

[brb3966-bib-0005] Dikmen, S. , McLean Jr, A. , Temkin, N. R. , & Wyler, A. R. (1986). Neuropsychologic outcome at one‐month postinjury. Archives of Physical Medicine and Rehabilitation, 67(8), 507–513. 3741074

[brb3966-bib-0006] Duncan, C. C. , Barry, R. J. , Connolly, J. F. , Fischer, C. , Michie, P. T. , Naatanen, R. , … Van Petten, C. (2009). Event‐related potentials in clinical research: Guidelines for eliciting, recording, and quantifying mismatch negativity, P300, and N400. Clinical Neurophysiology, 120(11), 1883–1908. https://doi.org/10.1016/j.clinph.2009.07.045 19796989 10.1016/j.clinph.2009.07.045

[brb3966-bib-0007] Duncan, C. C. , Summers, A. C. , Perla, E. J. , Coburn, K. L. , & Mirsky, A. F. (2011). Evaluation of traumatic brain injury: Brain potentials in diagnosis, function, and prognosis. International Journal of Psychophysiology, 82(1), 24–40. https://doi.org/10.1016/j.ijpsycho.2011.02.013 21356253 10.1016/j.ijpsycho.2011.02.013

[brb3966-bib-0008] Dymowski, A. R. , Ponsford, J. L. , & Willmott, C. (2016). Cognitive training approaches to remediate attention and executive dysfunction after traumatic brain injury: A single‐case series. Neuropsychological Rehabilitation, 26(5–6), 866–894. https://doi.org/10.1080/09602011.2015.1102746 26493353 10.1080/09602011.2015.1102746

[brb3966-bib-0009] Erdodi, L. A. , Roth, R. M. , Kirsch, N. L. , Lajiness‐O'neill, R. , & Medoff, B. (2014). Aggregating validity indicators embedded in Conners’ CPT‐II outperforms individual cutoffs at separating valid from invalid performance in adults with traumatic brain injury. Archives of Clinical Neuropsychology, 29(5), 456–466. 24957927 10.1093/arclin/acu026

[brb3966-bib-0010] Fonov, V. , Evans, A. C. , Botteron, K. , Almli, C. R. , McKinstry, R. C. , & Collins, D. L. (2011). Unbiased average age‐appropriate atlases for pediatric studies. NeuroImage, 54(1), 313–327. https://doi.org/10.1016/j.neuroimage.2010.07.033 20656036 10.1016/j.neuroimage.2010.07.033 PMC2962759

[brb3966-bib-0011] Gross, J. , Schmitz, F. , Schnitzler, I. , Kessler, K. , Shapiro, K. , Hommel, B. , & Schnitzler, A. (2004). Modulation of long‐range neural synchrony reflects temporal limitations of visual attention in humans. Proceedings of the National Academy of Sciences of the United States of America, 101(35), 13050–13055. https://doi.org/10.1073/pnas.0404944101 15328408 10.1073/pnas.0404944101 PMC516515

[brb3966-bib-0012] Guan, M. , Liao, Y. , Ren, H. , Wang, X. , Yang, Q. , Liu, X. , & Wang, W. (2015). Impaired response inhibition in juvenile delinquents with antisocial personality characteristics: A preliminary ERP study in a Go/Nogo task. Neuroscience Letters, 603, 1–5. https://doi.org/10.1016/j.neulet.2015.06.062 26189594 10.1016/j.neulet.2015.06.062

[brb3966-bib-0013] Halperin, J. M. , McKay, K. E. , & Newcorn, J. H. (2002). Development, reliability, and validity of the children's aggression scale‐parent version. Journal of the American Academy of Child and Adolescent Psychiatry, 41(3), 245–252. https://doi.org/10.1097/00004583-200203000-00003 11886018 10.1097/00004583-200203000-00003

[brb3966-bib-0014] Ham, T. E. , Bonnelle, V. , Hellyer, P. , Jilka, S. , Robertson, I. H. , Leech, R. , & Sharp, D. J. (2014). The neural basis of impaired self‐awareness after traumatic brain injury. Brain, 137(Pt 2), 586–597. https://doi.org/10.1093/brain/awt350 24371217 10.1093/brain/awt350 PMC3914476

[brb3966-bib-0015] Huang, L. Y. , She, H. C. , Chou, W. C. , Chuang, M. H. , Duann, J. R. , & Jung, T. P. (2013). Brain oscillation and connectivity during a chemistry visual working memory task. International Journal of Psychophysiology, 90(2), 172–179. https://doi.org/10.1016/j.ijpsycho.2013.07.001 23850831 10.1016/j.ijpsycho.2013.07.001

[brb3966-bib-0016] Iwanaga, M. , Kato, N. , Okazaki, T. , & Hachisuka, K. (2015). Effects of low‐dose milnacipran on event‐related potentials and neuropsychological tests in persons with traumatic brain injury: A preliminary study. Brain Injury, 29, 1252–1257. 10.3109/02699052.2015.1035332 26083047

[brb3966-bib-0017] Jacobs, B. , Beems, T. , Stulemeijer, M. , van Vugt, A. B. , van der Vliet, T. M. , Borm, G. F. , & Vos, P. E. (2010). Outcome prediction in mild traumatic brain injury: Age and clinical variables are stronger predictors than CT abnormalities. Journal of Neurotrauma, 27(4), 655–668. https://doi.org/10.1089/neu.2009.1059 20035619 10.1089/neu.2009.1059

[brb3966-bib-0018] Johnstone, S. J. , Barry, R. J. , & Clarke, A. R. (2013). Ten years on: A follow‐up review of ERP research in attention‐deficit/hyperactivity disorder. Clinical Neurophysiology, 124(4), 644–657. https://doi.org/10.1016/j.clinph.2012.09.006 23063669 10.1016/j.clinph.2012.09.006

[brb3966-bib-0019] Kay, T. , Harrington, D. E. , Adams, R. , Anderson, T. , Berrol, S. , Cicerone, K. , … Horn, L. (1993). Definition of mild traumatic brain injury. The Journal of Head Trauma Rehabilitation, 8, 86–87.

[brb3966-bib-0020] Klein, A. , & Tourville, J. (2012). 101 labeled brain images and a consistent human cortical labeling protocol. Frontiers in Neuroscience, 6, 171. 23227001 10.3389/fnins.2012.00171 PMC3514540

[brb3966-bib-0021] Klimesch, W. , Sauseng, P. , & Hanslmayr, S. (2007). EEG alpha oscillations: The inhibition‐timing hypothesis. Brain Research Reviews, 53(1), 63–88. https://doi.org/10.1016/j.brainresrev.2006.06.003 16887192 10.1016/j.brainresrev.2006.06.003

[brb3966-bib-0022] Larson, M. J. , Clayson, P. E. , & Farrer, T. J. (2012). Performance monitoring and cognitive control in individuals with mild traumatic brain injury. Journal of the International Neuropsychological Society, 18(2), 323–333. https://doi.org/10.1017/S1355617711001779 22272692 10.1017/S1355617711001779

[brb3966-bib-0023] Larson, M. J. , Kaufman, D. A. , Schmalfuss, I. M. , & Perlstein, W. M. (2007). Performance monitoring, error processing, and evaluative control following severe TBI. Journal of the International Neuropsychological Society, 13(6), 961–971. 17942014 10.1017/S1355617707071305

[brb3966-bib-0024] Lee, C. N. , Koh, Y. C. , Moon, C. T. , Park, D. S. , & Song, S. W. (2015). Serial mini‐mental status examination to evaluate cognitive outcome in patients with traumatic brain injury. Korean Journal of Neurotrauma, 11(1), 6–10. https://doi.org/10.13004/kjnt.2015.11.1.6 27169058 10.13004/kjnt.2015.11.1.6 PMC4847490

[brb3966-bib-0025] Lee, J. Y. , Lindquist, K. A. , & Nam, C. S. (2017). Emotional granularity effects on event‐related brain potentials during affective picture processing. Frontiers in Human Neuroscience, 11, 133. 28392761 10.3389/fnhum.2017.00133 PMC5364149

[brb3966-bib-0026] Mannarelli, D. , Pauletti, C. , Grippo, A. , Amantini, A. , Augugliaro, V. , Curra, A. , … Fattapposta, F. (2015). The role of the right dorsolateral prefrontal cortex in phasic alertness: Evidence from a contingent negative variation and repetitive transcranial magnetic stimulation study. Neural Plasticity, 2015, 410785. 26090234 10.1155/2015/410785 PMC4458283

[brb3966-bib-0027] Mathias, J. L. , & Wheaton, P. (2007). Changes in attention and information‐processing speed following severe traumatic brain injury: A meta‐analytic review. Neuropsychology, 21(2), 212–223. https://doi.org/10.1037/0894-4105.21.2.212 17402821 10.1037/0894-4105.21.2.212

[brb3966-bib-0028] Meeuwissen, E. B. , Takashima, A. , Fernandez, G. , & Jensen, O. (2011). Increase in posterior alpha activity during rehearsal predicts successful long‐term memory formation of word sequences. Human Brain Mapping, 32(12), 2045–2053. https://doi.org/10.1002/hbm.21167 21162031 10.1002/hbm.21167 PMC6870165

[brb3966-bib-0029] Mognon, A. , Jovicich, J. , Bruzzone, L. , & Buiatti, M. (2011). ADJUST: An automatic EEG artifact detector based on the joint use of spatial and temporal features. Psychophysiology, 48(2), 229–240. https://doi.org/10.1111/j.1469-8986.2010.01061.x 20636297 10.1111/j.1469-8986.2010.01061.x

[brb3966-bib-0030] Nam, C. S. , Jeon, Y. , Kim, Y. J. , Lee, I. , & Park, K. (2011). Movement imagery‐related lateralization of event‐related (de)synchronization (ERD/ERS): Motor‐imagery duration effects. Clinical Neurophysiology, 122(3), 567–577. https://doi.org/10.1016/j.clinph.2010.08.002 20800538 10.1016/j.clinph.2010.08.002

[brb3966-bib-0031] Nicholls, C. , Bruno, R. , & Matthews, A. (2015). Chronic cannabis use and ERP correlates of visual selective attention during the performance of a flanker go/nogo task. Biological Psychology, 110, 115–125. https://doi.org/10.1016/j.biopsycho.2015.07.013 26232619 10.1016/j.biopsycho.2015.07.013

[brb3966-bib-0032] Olofsson, J. K. , & Polich, J. (2007). Affective visual event‐related potentials: Arousal, repetition, and time‐on‐task. Biological Psychology, 75(1), 101–108. https://doi.org/10.1016/j.biopsycho.2006.12.006 17275979 10.1016/j.biopsycho.2006.12.006 PMC1885422

[brb3966-bib-0033] Padrao, G. , Rodriguez‐Herreros, B. , Perez Zapata, L. , & Rodriguez‐Fornells, A. (2015). Exogenous capture of medial‐frontal oscillatory mechanisms by unattended conflicting information. Neuropsychologia, 75, 458–468. https://doi.org/10.1016/j.neuropsychologia.2015.07.004 26151855 10.1016/j.neuropsychologia.2015.07.004

[brb3966-bib-0034] Palva, S. , & Palva, J. M. (2007). New vistas for alpha‐frequency band oscillations. Trends in Neurosciences, 30(4), 150–158. https://doi.org/10.1016/j.tins.2007.02.001 17307258 10.1016/j.tins.2007.02.001

[brb3966-bib-0035] Pfurtscheller, G. , & Lopes da Silva, F. H. (1999). Event‐related EEG/MEG synchronization and desynchronization: Basic principles. Clinical Neurophysiology, 110(11), 1842–1857. https://doi.org/10.1016/S1388-2457(99)00141-8 10576479 10.1016/s1388-2457(99)00141-8

[brb3966-bib-0036] Poch, C. , Campo, P. , & Barnes, G. R. (2014). Modulation of alpha and gamma oscillations related to retrospectively orienting attention within working memory. The European Journal of Neuroscience, 40(2), 2399–2405. https://doi.org/10.1111/ejn.12589 24750388 10.1111/ejn.12589 PMC4215597

[brb3966-bib-0037] Rabinowitz, A. R. , & Levin, H. S. (2014). Cognitive sequelae of traumatic brain injury. The Psychiatric Clinics of North America, 37(1), 1–11. https://doi.org/10.1016/j.psc.2013.11.004 24529420 10.1016/j.psc.2013.11.004 PMC3927143

[brb3966-bib-0038] Ranganath, C. , & Rainer, G. (2003). Neural mechanisms for detecting and remembering novel events. Nature Reviews Neuroscience, 4(3), 193–202. https://doi.org/10.1038/nrn1052 12612632 10.1038/nrn1052

[brb3966-bib-0039] Schmitter‐Edgecombe, M. , & Robertson, K. (2015). Recovery of visual search following moderate to severe traumatic brain injury. Journal of Clinical and Experimental Neuropsychology, 37(2), 162–177. https://doi.org/10.1080/13803395.2014.998170 25671675 10.1080/13803395.2014.998170 PMC4355332

[brb3966-bib-0040] Segalowitz, S. J. , Dywan, J. , & Unsal, A. (1997). Attentional factors in response time variability after traumatic brain injury: An ERP study. Journal of the International Neuropsychological Society, 3(2), 95–107. 9126851

[brb3966-bib-0041] Shumskaya, E. , Andriessen, T. M. , Norris, D. G. , & Vos, P. E. (2012). Abnormal whole‐brain functional networks in homogeneous acute mild traumatic brain injury. Neurology, 79(2), 175–182. https://doi.org/10.1212/WNL.0b013e31825f04fb 22744656 10.1212/WNL.0b013e31825f04fb

[brb3966-bib-0042] Smits, M. , Dippel, D. W. , Steyerberg, E. W. , de Haan, G. G. , Dekker, H. M. , Vos, P. E. , … Hunink, M. G. (2007). Predicting intracranial traumatic findings on computed tomography in patients with minor head injury: The CHIP prediction rule. Annals of Internal Medicine, 146(6), 397–405. https://doi.org/10.7326/0003-4819-146-6-200703200-00004 17371884 10.7326/0003-4819-146-6-200703200-00004

[brb3966-bib-0043] von Stein, A. , Rappelsberger, P. , Sarnthein, J. , & Petsche, H. (1999). Synchronization between temporal and parietal cortex during multimodal object processing in man. Cerebral Cortex, 9(2), 137–150. 10220226 10.1093/cercor/9.2.137

[brb3966-bib-0044] Tallon‐Baudry, C. , Bertrand, O. , & Fischer, C. (2001). Oscillatory synchrony between human extrastriate areas during visual short‐term memory maintenance. The Journal of Neuroscience, 21(20), Rc177. 11588207 10.1523/JNEUROSCI.21-20-j0008.2001 PMC6763859

[brb3966-bib-0045] Uhlhaas, P. J. , Haenschel, C. , Nikolic, D. , & Singer, W. (2008). The role of oscillations and synchrony in cortical networks and their putative relevance for the pathophysiology of schizophrenia. Schizophrenia Bulletin, 34(5), 927–943. https://doi.org/10.1093/schbul/sbn062 18562344 10.1093/schbul/sbn062 PMC2632472

[brb3966-bib-0046] Wen, H. B. , Zhang, Z. X. , Niu, F. S. , & Li, L. (2008). The application of Montreal cognitive assessment in urban Chinese residents of Beijing. Zhonghua Nei Ke Za Zhi, 47(1), 36–39. 18346324

[brb3966-bib-0047] Wu, J. , Yuan, Y. , Cao, C. , Zhang, K. , Wang, L. , & Zhang, L. (2015). The relationship between response inhibition and posttraumatic stress symptom clusters in adolescent earthquake survivors: An event‐related potential study. Scientific Reports, 5, 8844 https://doi.org/10.1038/srep08844 25740732 10.1038/srep08844 PMC4350076

[brb3966-bib-0048] Zhou, Y. , Milham, M. P. , Lui, Y. W. , Miles, L. , Reaume, J. , Sodickson, D. K. , … Ge, Y. (2012). Default‐mode network disruption in mild traumatic brain injury. Radiology, 265(3), 882–892. https://doi.org/10.1148/radiol.12120748 23175546 10.1148/radiol.12120748 PMC3504316

[brb3966-bib-0049] Zygierewicz, J. , Durka, P. J. , Klekowicz, H. , Franaszczuk, P. J. , & Crone, N. E. (2005). Computationally efficient approaches to calculating significant ERD/ERS changes in the time‐frequency plane. Journal of Neuroscience Methods, 145(1–2), 267–276. https://doi.org/10.1016/j.jneumeth.2005.01.013 15922042 10.1016/j.jneumeth.2005.01.013

